# A rare focal atrial tachycardia arising from the proximal middle cardiac vein: a case report

**DOI:** 10.1186/s12872-023-03172-4

**Published:** 2023-03-29

**Authors:** Min Guo, Nan Zhang, Gao Jia, Guijin Ma, Xin Li, Rui Wang

**Affiliations:** Department of Cardiology, First Hospital of Shanxi Medical University, No.85 Jiefang Road, Taiyuan, 030001 Shanxi China

**Keywords:** Case report, Focal atrial tachycardia, Radiofrequency ablation, Catheter ablation, Proximal middle cardiac vein

## Abstract

**Background:**

Focal atrial tachycardia (FAT) always originates from atrium specific sites and can be successfully cured by radiofrequency (RF) ablation. However, the middle cardiac vein (MCV) is a rare site of focal atrial tachycardia. Herein, we present a case of a 20-year-old young woman with FAT. Electrophysiological examination showed FAT arising from the proximal middle cardiac vein (pMCV), and successful RF ablation was applied with a low power and short-ablation.

**Case presentation:**

A 20-year-old woman with no structural heart disease suffered recurrent supraventricular tachycardia for 1 year. Physical examination, laboratory studies and the echocardiography results of this patient were normal. A 12-lead electrocardiogram (ECG) showed a narrow QRS and long RP tachycardia which was always triggered by a sinus rhythm. The patient underwent an electrophysiological study and found the earliest activation was in the proximal MCV (pMCV). After a low power and short-ablation, AT was terminated and noninducible by programmed pacing with or without isoproterenol infusion.

**Conclusion:**

This case presented a rare case of FAT arising from the pMCV. We demonstrate that low power and short-ablation are effective in AT arising from specific areas such as the coronary sinus ostium and pMCV.

## Background

Focal atrial tachycardia (FAT) is an uncommon supraventricular tachycardia disease, and most of it originates from left and right atrium specific sites, such as crista terminals [1, 2], near the mitral annulus and tricuspid annulus [3, 4], coronary sinus ostium [5], and pulmonary veins [6]. To our knowledge, the middle cardiac vein (MCV) is a rare site of focal atrial tachycardia. Herein, we present a case of successful radiofrequency (RF) ablation of FAT arising from the proximal MCV (pMCV).

## Case presentation

**Fig. 1:**
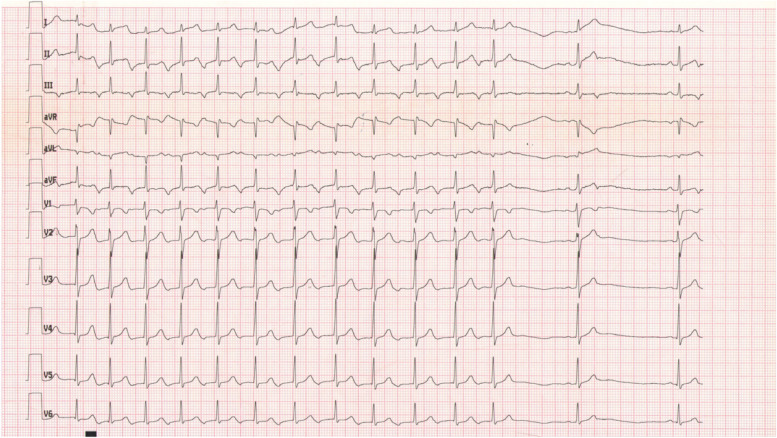
12-lead electrocardiogram (ECG) showing narrow QRS and long RP tachycardia with 1:1 atrioventricular conduction

A 20-year-old woman with no structural heart disease suffered recurrent supraventricular tachycardia nonresponsive to beta-blocker therapy. A 12-lead electrocardiogram (ECG) showed a narrow QRS and long RP tachycardia at a rate of 80–100 beats per minute (bpm). Event monitoring revealed short runs of tachycardia at 110–125 bpm lasting for 2–20 beats. The tachycardia was always triggered by a sinus rhythm and the P-wave morphology on 12-lead ECG was inverted in inferior leads, positive in I, avL and biphasic in V1 (Fig. 1). Physical examination, laboratory studies and the echocardiography results of this patient were normal.

**Fig. 2:**
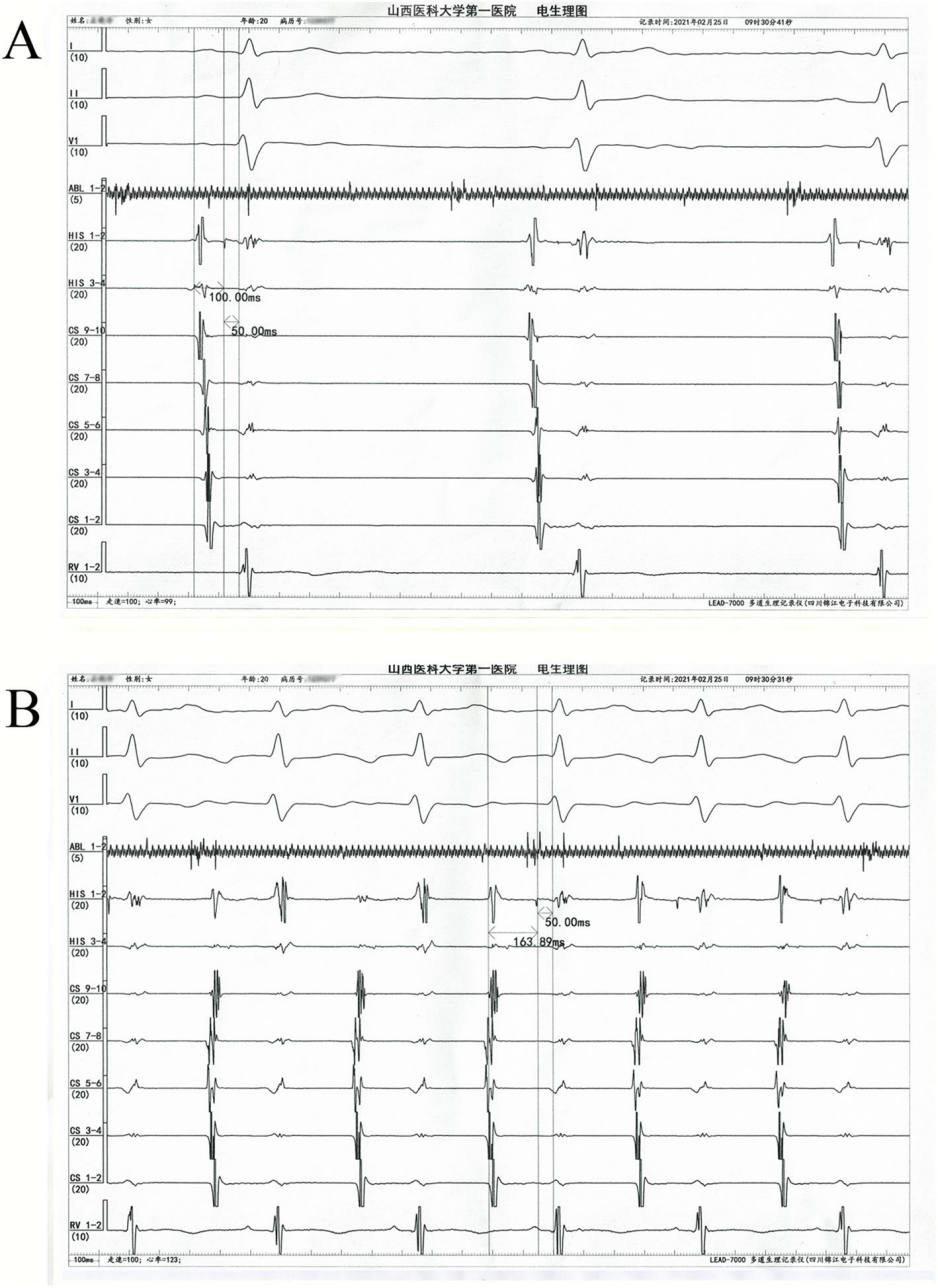
Intracardiacelectrogram revealed a different atrial activation from the sinus rhythm, being 5–6 poles is the earliest activation and the measurements made in tachycardia were AH of 164 ms and HV of 50 ms. **A** The basal measurements made in sinus rhythm were AH of 100 ms and HV of 50 ms (**B**)

**Fig. 3:**
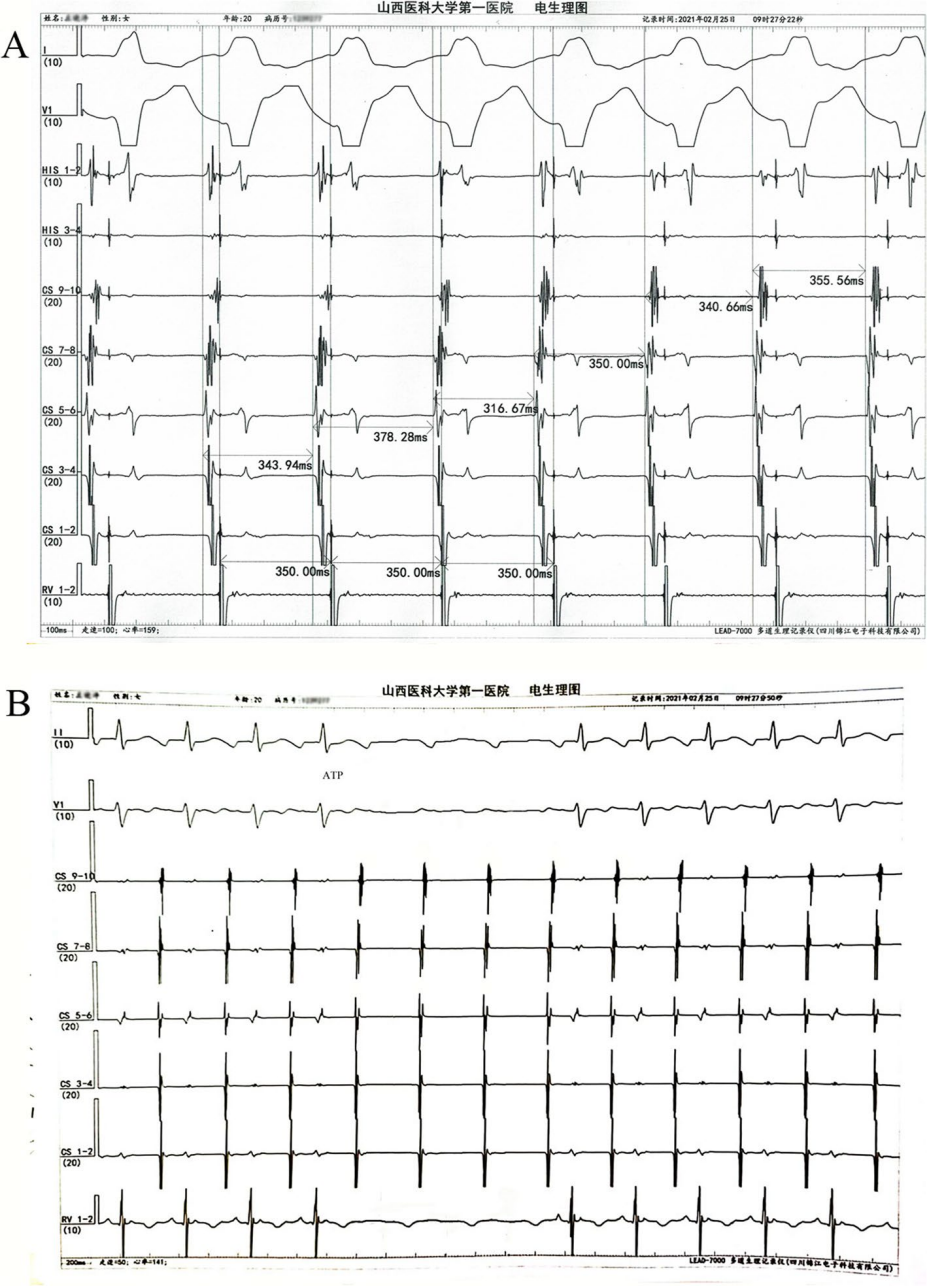
Electrophysiological study. **A** Ventricular overdrive pacing (VOP) at 350 ms during tachycardia was performed, and an atrioventricular dissociation was observed. **B** The tachycardia does not terminate after intravenous adenosine inducing an atrioventricular block

**Fig. 4:**
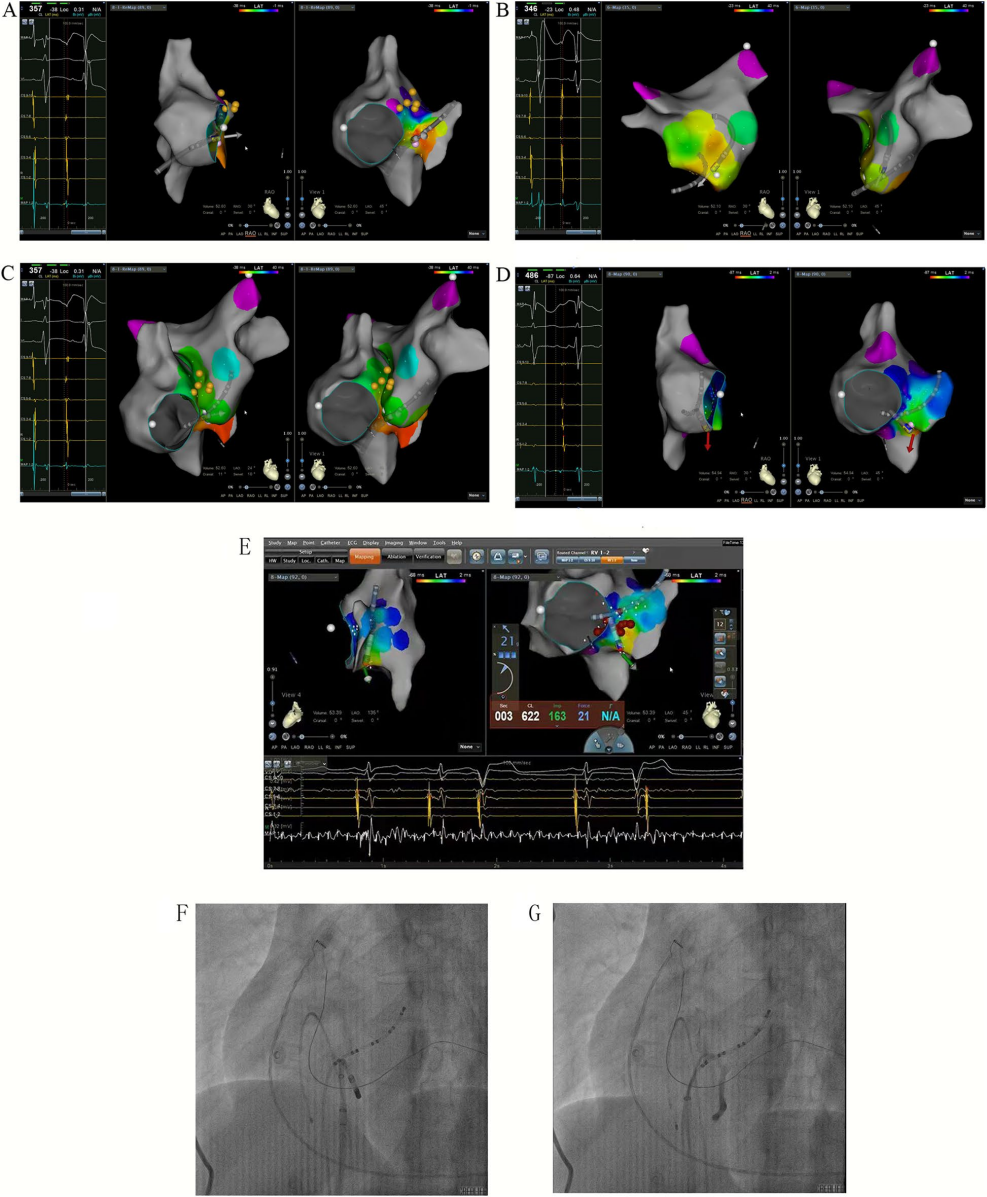
Three-dimensional (3D) activation map. **A** A ThermoCool SmartTouch catheter was used for the right atrium mapping, revealing 5–6 poles of the decapolar catheter as the earliest activation. **B** and **C** Mapping of the left atrium found no earlier atrial activity in the left posterior septum. **D** The earliest activation was near the site of the proximal middle cardiac vein (pMCV). There were low-amplitude, fractionated electrograms at the earliest activation site. **E** Three-dimensional image of RF ablation. **F** X-ray image of RF ablation (LAO fluoroscopic view). **G** Angiography through the multifunctional catheter confirmed that this successful ablation site was in the proximal MCV (LAO fluoroscopic view)

**Fig. 5:**
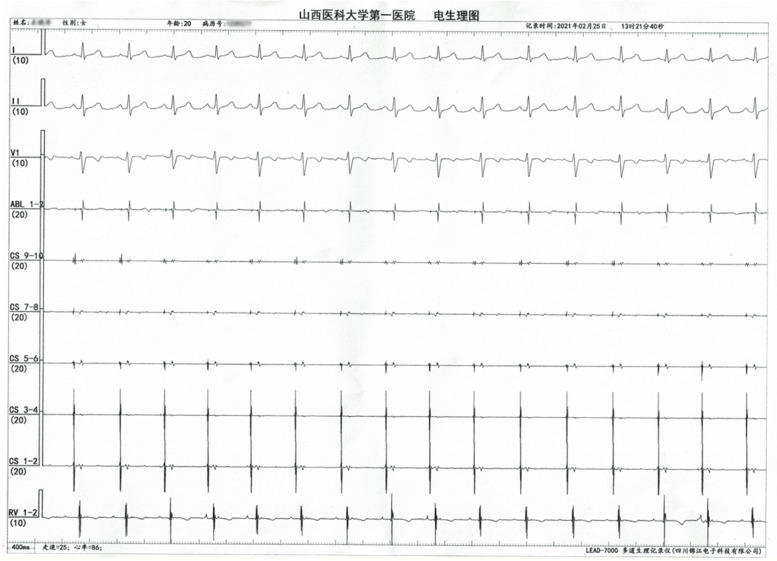
12-lead electrocardiogram (ECG) after RF ablation

After the patient’s family had signed informed consent for our following ablation procedure treatment policy, the patient underwent an electrophysiological study and subsequent radiofrequency ablation using three-dimensional electroanatomical mapping system (Carto3, Biosense Webster, Diamond Bar, CA, USA). Multipolar catheters were placed in the His-bundle region, right ventricle and coronary sinus. Tachycardia occurred and terminated spontaneously with a tachycardia cycle length between 370–400 ms and 1:1 atrioventricular conduction. The decapolar catheter in the coronary sinus revealed a different atrial activation from the sinus rhythm with 5–6 poles being the earliest activation. The basal measurements made in sinus rhythm were AH of 100 ms and HV of 50 ms while the measurements made in tachycardia were AH of 164 ms and HV of 50 ms (Fig. 2A and B). We performed ventricular overdrive pacing (VOP) at a 30 ms cycle length less than the tachycardia cycle lengths (TCLs) during tachycardia, and atrioventricular dissociation was observed, which could suggest AT (Fig. 3A). In addition, intravenous adenosine that caused an atrioventricular block failed to terminate the tachycardia confirming AT again (Fig. 3B). A ThermoCool SmartTouch catheter with a 3.5 mm open-irrigated-tip electrode (Biosense Webster, Diamond Bar, CA, USA) was used for right atrium mapping, which revealed that the earliest endocardial activation was located in the posterior septum. However, AT could not be terminated by fractionated RF ablation at the right atrium and coronary sinus ostium. Then, mapping of the left atrium performed by the transseptal puncture approach found no earlier atrial activity in the left posterior septum. Finally, we remapped the right posterior septum and coronary sinus ostium, and found that the earliest activation was near the site of the proximal middle cardiac vein (pMCV). There were low-amplitude, fractionated electrograms ahead of P wave onset by 24 ms (Fig. 4A-D) at the earliest activation site. In addition, angiography through the multifunctional catheter confirmed that this earliest site was in the pMCV (Fig. 4E-G). AT was successfully terminated within 5 s after a single RF ablation with a power set at 30 W and 55 °C with 30 ml/min irrigation for 20 s. After RF ablation, AT was noninducible by programmed pacing with or without isoproterenol infusion (Fig. 5). Because a low power and short-ablation duration may lead to an increase in the possibility of recurrence, we followed up for 1 month and found no AT recurred in this patient.

## Discussion and conclusion

Previous studies have demonstrated that AT can be divided into FAT and macro-reentrant AT. Patients with FAT are problematic owing to their poor response to medical treatment [7]. RF ablation has been well-recognized as a better treatment choice in patients with FAT [1, 3–9]. The CS is a cardiac venous structure that has two systems of drainage, including the left ventricular veins and the middle cardiac vein. MCV always drains the posterior aspect of the left ventricle. Although the ostium of CS is recognized as an uncommon origin site of FAT (6.7%), a few FATs have been reported to originate from the MCV. Our case report provides evidence that pMCV serves as an arrhythmogenic substrate to drive FAT.

In the present case, tachycardia always started during sinus rhythm, and thus we thought that it may be permanent junctional reciprocating tachycardia (PJRT) from the characteristic of the ECG. However, the response to VOP and intravenous adenosine demonstrated the diagnosis of FAT. The earliest site of activation was then mapped to the coronary sinus ostium, which was also consistent with the ECG, with P-waves deeply negative in all inferior leads, negative or isoelectric becoming positive in lead V1, and then progressively negative across the precordium [5]. In addition, further mapping confirmed that the earliest activation was near the site of the pMCV. Previous studies have reported successful results with RF ablation in the coronary sinus ostium [5, 10]. As far as we known, MCV is a known anatomical structure that, instead of being an atrial structure, is more located over the LV rather than an atrial structure. The catheter located at this site usually shows a low-amplitude A wave and a relatively high-amplitude V wave, which similar close to LV activity. Ventricular arrhythmias have been more commonly described from the ventricular venous system; however, accessory pathways which are often successful ablated from atrial characterized with target potential of a low-amplitude A wave and a high-amplitude V wave have been also described in the MCV as well. The pathophysiology of AT origination in venous system has been linked to the presence of muscular sleeves, for example in proximal coronary sinus. However, catheter ablation of FAT originating from MCV has not yet been reported. Here, we demonstrated a unique FAT arising from the pMCV. Given the RF current delivery within a vein is associated with various complications, such as cardiac tamponade, stenosis, vessel occlusion, char formation or seam pop formation. Reasonable options for CS ablation reported in the literature are: 1) RF ablation with a low power set (20–30 W) and short duration for 10–20 s [11]; 2) cryothermal ablation [12]; 3) the percutaneous epicardial approach when there is endocardial inaccessibility [13]; 4) angiographic definition of the coronary sinus and coronary arteries may be helpful to avoid the complications associated with this site [14]. Finally, after confirming the catheter location in the pMCV by angiography, low-power short-duration ablation was applied, and the tachycardia was terminated by RF ablation.

In conclusion, we presented a rare case of FAT arising from the pMCV. We hypothesize that low power and short-ablation are effective in AT arising from specific areas such as the coronary sinus ostium and pMCV.

## Data Availability

The original contributions presented in the study can be inquired directed to the corresponding author.

## Abbreviations

FAT: Focal atrial tachycardia
MCV: Middle cardiac vein
RF: Radiofrequency
ECG: Electrocardiogram
VOP: Ventricular overdrive pacing
TCLs: Tachycardia cycle lengths
PJRT: Permanent junctional reciprocating tachycardia
